# TyrR, the regulator of aromatic amino acid metabolism, is required for mice infection of *Yersinia pestis*

**DOI:** 10.3389/fmicb.2015.00110

**Published:** 2015-02-12

**Authors:** Zhongliang Deng, Zizhong Liu, Junming He, Jing Wang, Yanfeng Yan, Xiaoyi Wang, Yujun Cui, Yujing Bi, Zongmin Du, Yajun Song, Ruifu Yang, Yanping Han

**Affiliations:** ^1^Department of Sanitary Inspection, School of Public Health, University of South ChinaHengyang, China; ^2^State Key Laboratory of Pathogen and Biosecurity, Beijing Institute of Microbiology and EpidemiologyBeijing, China; ^3^Animal Husbandry Base Teaching and Research Section, College of Animal Science and Technology, Hebei North UniversityZhangjiakou, China

**Keywords:** *Yersinia pestis*, TyrR, aromatic amino acid metabolism, pathogenesis

## Abstract

*Yersinia pestis*, the causative agent of plague, poses a serious health threat to rodents and human beings. TyrR is a transcriptional regulator (TyrR) that controls the metabolism of aromatic amino acids in *Escherichia coli*. In this paper, TyrR played an important role in *Y. pestis* virulence. Inactivation of *tyrR* did not seem to affect the *in vitro* growth of this organism, but resulted in at least 10,000-fold attenuation compared with the wild-type (WT) strain upon subcutaneous infection to mice. In addition, loads of *tyrR* mutant within mice livers and spleens significantly decreased compared with the WT strain. Transcriptome analysis revealed that TyrR, directly or indirectly, regulated 29 genes encoded on *Y. pestis* chromosome or plasmids under *in vitro* growth condition. Similar to the regulatory function of this protein in *E. coli*, five aromatic-pathway genes (*aroF-tyrA*, aroP, *aroL*, and *tyrP*) were significantly reduced upon deletion of the *tyrR* gene. Two genes (*glnL* and *glnG*) that encode sensory histidine kinase and regulator in a two-component regulatory system involved in nitrogen assimilation were downregulated in the *tyrR* mutant. Several genes encoding type III secretion proteins were transcribed by 2.0–4.2-fold in a *tyrR* mutant relative to the WT strain. Interestingly, the acid-stressed genes, *hdeB* and *hdeD*, were downregulated, and such downregulation partly accounted for the decrease in tolerance of the *tyrR* mutant under acidic conditions. In conclusion, regulation of TyrR in *Y. pestis* is similar to, but distinct from, that in *E. coli*. TyrR is a metabolic virulence determinant in *Y. pestis* that is important for extracellular survival and/or proliferation.

## Introduction

*Yersinia pestis* can cause fatal infections in rodents and humans and is usually transmitted by flea biting (Perry and Fetherston, 1997). A few virulence determinants have been defined based on their contributions to flea transmission colonization, invasion, intracellular growth or extracellular proliferation (Charnetzky and Shuford, 1985; Lindler et al., 1990; Galyov et al., 1991; Lahteenmaki et al., 1998). Numerous efforts to determine the mechanism of *Yersinia* pathogenesis are mainly focused on a few previously established virulence determinants (Perry and Fetherston, 1997). Many other genes encoding two-component systems or global transcriptional regulators have been proven to be implicated in the regulatory networks involved in *Y. pestis* pathogenicity (Oyston et al., 2000; Cathelyn et al., 2006; Zhan et al., 2008; Geng et al., 2009). More than 30 genes responsible for critical metabolic pathways functioned during *Y. pestis* fitness *in vivo* (Palace et al., 2014). *Y. pestis* lacking ~47-kb DNA fragment that contains more than 40 genes was obtained by our laboratory. Surprisingly, we observed the significantly attenuated virulence of this mutant via subcutaneous infection in mice. This observation prompted us to investigate which gene or operon was responsible for such attenuation. After careful tracing via gene knockout within the 47-kb region, a transcriptional regulator (TyrR) was confirmed to be mainly responsible for the virulence phenotype. A number of DNA-binding regulators could control gene expression common to many bacterial species. TyrR is responsible for aromatic amino acids metabolism in *Escherichia coli*, which is evolutionarily acquired and is only present in γ-proteobacteria (Panina et al., 2001; Song et al., 2005). Subsequent evidence showed that the recruitment of structure genes by TyrR is dynamic and is evolving further (Pittard et al., 2005). Here we showed that TyrR is required for *Y. pestis* pathogenesis and extracellular survival/proliferation. Transcriptome analysis demonstrated that the regulation of TyrR in *Y. pestis* is similar to, but distinct from, that in *E. coli*.

## Experimental procedure

### Bacterial strains

*Y. pestis* wild-type (WT) strain 201, 47-kb fragment and *tyrR* deletion mutant were used in this study. Strain 201 was isolated from *Microtus brandti* in Inner Mongolia, China. Strain 201 is supposed to be avirulent to humans, but highly lethal to mice (Fan et al., 1995). The deletion mutant of *Y. pestis* was constructed by replacing the entire target gene with the *kan* cassette by using λ-Red homologos recombination. To obtain a strain in which Δ*tyrR* is complemented, plasmid pACYC184, which contains a PCR fragment covering a region from 400 bp fragment upstream to 100 bp downstream of the *tyrR* gene, was introduced into *Y. pestis* Δ*tyrR*.

### Determination of bacterial growth curves *in vitro*

*Y. pestis* strains were grown in LB medium at 26°C to exponential phase (OD_620_ ≈ 1.0). The bacterial cultures were diluted 1:20 in LB medium with the indicated pH value and incubated at 26°C. Bacterial growth was monitored by measuring absorbance at OD_620_. The experiments were performed in three independent cultures. Results were expressed as the mean percentage ± standard deviation from the three independent experiments.

### Mouse infection

*Y. pestis* strains were grown in brain-heart infusion (BHI) broth at 26°C to OD_620_ ≈ 1.0. Bacterial cells were harvested by centrifugation, washed twice and resuspended in phosphate-buffered saline (PBS). Groups of five or six female BALB/c mice (6-weeks-old) were injected subcutaneously with serial dilutions of bacterial cultures. Mortality was recorded daily for 14 d. The 50% lethal dose (LD_50_) was calculated by the Reed-Muench equation (Reed and Muench, 1938).

To monitor bacterial growth dynamics *in vivo*, *Y. pestis* WT strain 201 and the *tyrR* deletion mutant grown to exponential phase were washed and diluted to 600 CFU/mL in PBS. A 1:1 mixture (100 μL) of the two bacterial strains was used to infect eight BALB/c mice intravenously. For competitive index (CI) determination, the infected mice were sacrificed at 48 and 72 h postinfection, and livers and spleens were removed for homogenization. Bacterial loads per organ were determined by calculating the number of viable bacteira of the resulting homogenates plated on Hottinger agar with and without kanamycin. The CI value was calculated as the ratio of the number of mutant/WT bacteria recovered. The data were analyzed by Student's *t*-test, with *P* < 0.05 considered statistically significant. All mouse experiments were carried out according to the Guidelines for the Welfare and Ethics of Laboratory Animals of Beijing.

### Transcription analysis by using RNA-seq and real-time PCR

*Y. pestis* WT strain 201 and the Δ*tyrR* mutant were grown at 26°C in BHI medium to middle exponential phase and then transferred to 37°C for 3 h. Total RNA was extracted using the TRIzol Reagent according to the manufacturer's protocol (Invitrogen). These two RNA samples were subjected to cDNA library construction and deep sequencing performed by LC Sciences LLC, USA, as previously described (Yan et al., 2013). RPKM was used to estimate the expression levels of mRNA transcripts encoded by *Y. pestis* chromosome and plasmids. Those genes with cDNA coverage below 20 in both samples were removed from further analysis. The fold change of mRNA expression levels between WT and *tyrR* mutant were calculated. Two-fold was used as threshold to determine the differentially regulated genes.

For quantitative RT-PCR, cDNA was generated using 5 μg of total RNA and 3 μg of random hexamer primers with the Superscript II system. All primer pairs produced a 150–200 nt amplicon when *Y. pestis* genomic DNA was used as the template for PCR. Real-time PCR was performed in duplicate for each RNA preparation using the LightCycler system (Roche) with an appropriate dilution of cDNA as a template. Based on the standard curve of 16S rRNA, the relative mRNA level was determined by calculating the threshold cycle (ΔCt) of each gene by the classic ΔCt method. Quantification of 16S rRNA was also used to normalize the values of all the other genes in the RT-PCR experiment.

### Measurement of secreted LcrV protein by using western blotting

*Y. pestis* WT strain, the Δ*tyrR* mutant and the *tyrR* complementary strain were grown at 26°C in TMH medium without calcium to OD_620_ = 1.0 and then transferred to 37°C for 3 h. Secreted proteins from bacterial supernatants were precipitated with trichloroacetic acid (TCA). Proteins from equal amount of bacteria were separated on SDS–PAGE and immunoblotted with LcrV polyclonal antibody followed by detection using the Odyssey Infrared Imaging System.

## Results

### TyrR is required for *Y. pestis* infection upon subcutaneous inoculation to mice

**Figure 1:**
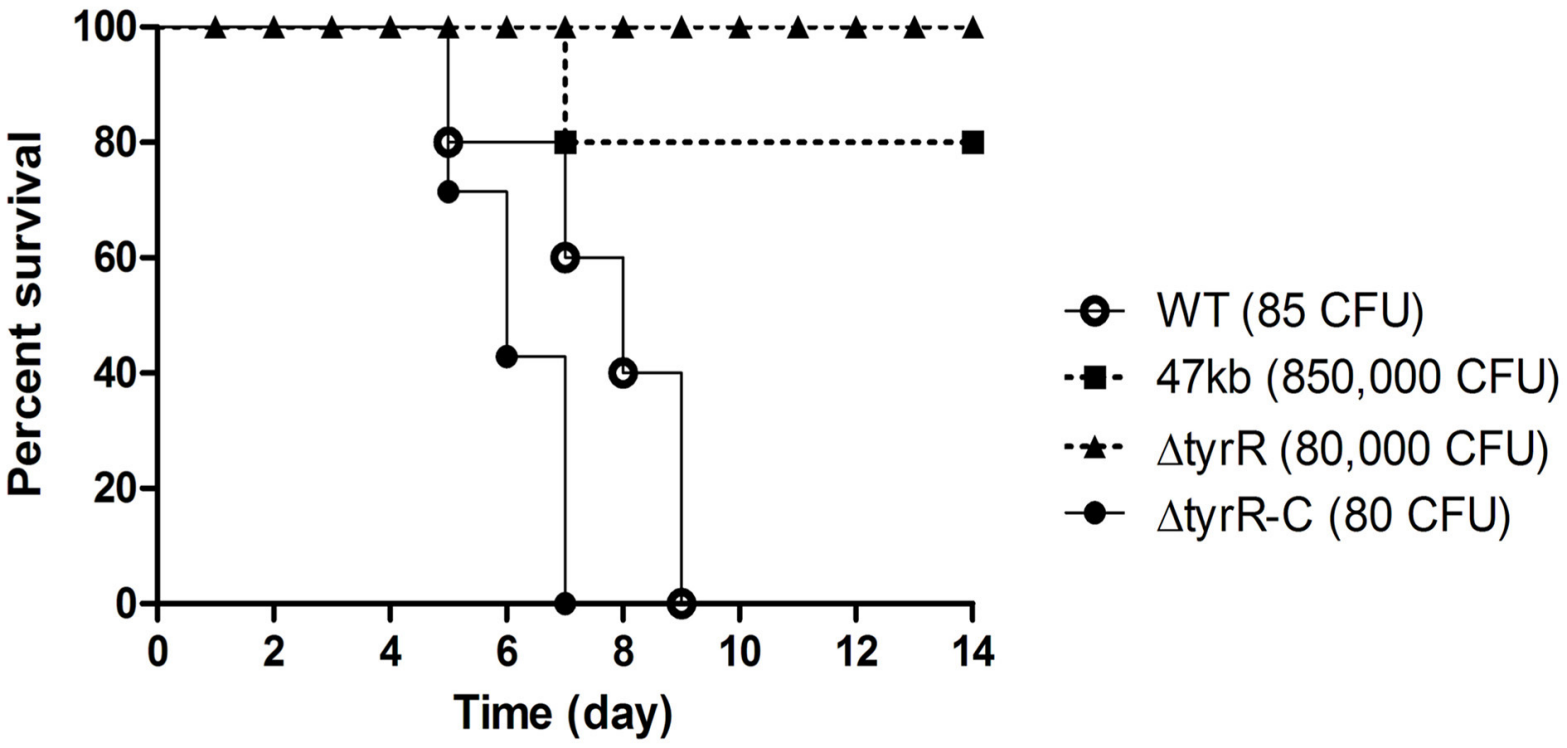
**Effect of the *tyrR* gene on *Y. pestis* pathogenesis in mice**. Groups of five or seven BALB/c mice were inoculated by subcutaneous injection with appropriated dose of *Y. pestis* strain 201 (open circles), 47-kb mutant (filled diamonds), *tyrR* (filled triangles) or *tyrR* complementary strain (filled circles). Mice mortality was monitored for 14 d.

We observed the strong attenuation of a deletion mutant of ~47-kb DNA fragment containing more than 40 genes in BALB/c mice upon subcutaneous inoculation. To trace which gene(s) or operon is responsible for the attenuated phenotype, DNA fragments within the 47-kb region were knockout one by one from the WT strain. The resulting mutants were subject to mice infection, respectively. Finally, a ~8-kb fragment containing *tyrR* and its flanking sequences were confirmed to be the main reason for the virulence phenotype. Therefore, we decided to assess the roles of TyrR protein in *Y. pestis* pathogenesis. Mice were infected subcutaneously (*s.c*.) with increasing numbers of WT, *tyrR* mutant, and complementary strain to estimate the virulence by calculating LD_50_. The LD_50_ of both the WT strain 201 and the *tyrR* complementary strain was, <8 CFU, but up to about 8 × 10^4^ cells of the *tyrR* mutant was not lethal to *s.c*. inoculated mice (Figure 1). The observation suggested that the significantly attenuated virulence of the *tyrR* mutant is due to the lack of TyrR protein rather than to polar effects caused by the insertion of a kanamycin resistance cassette.

### TyrR contributes to growth dynamics *in vivo*

**Figure 2:**
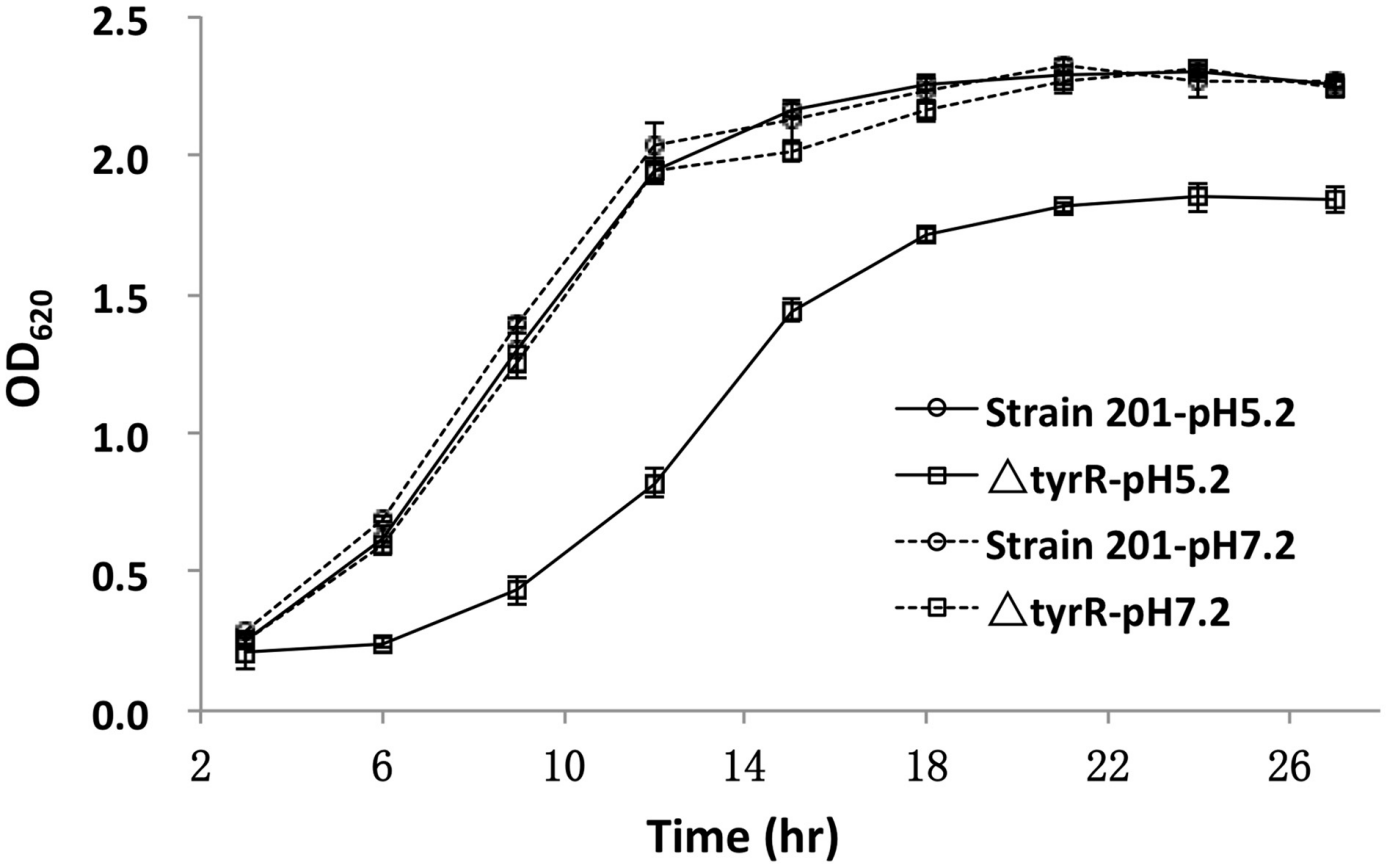
**Growth curves of the WT and *tyrR* mutant strains under two different conditions**. Overnight cultures of the WT strain 201 and *tyrR* mutant were used to inoculate fresh LB broth with pH 5.2 or 7.0. The OD_620_ values were recorded at fixed time points. Each time point is the average of two measurements and error bars represent standard deviations. The vertical arrows indicate the time point at which samples were removed for RNA extraction.

**Figure 3:**
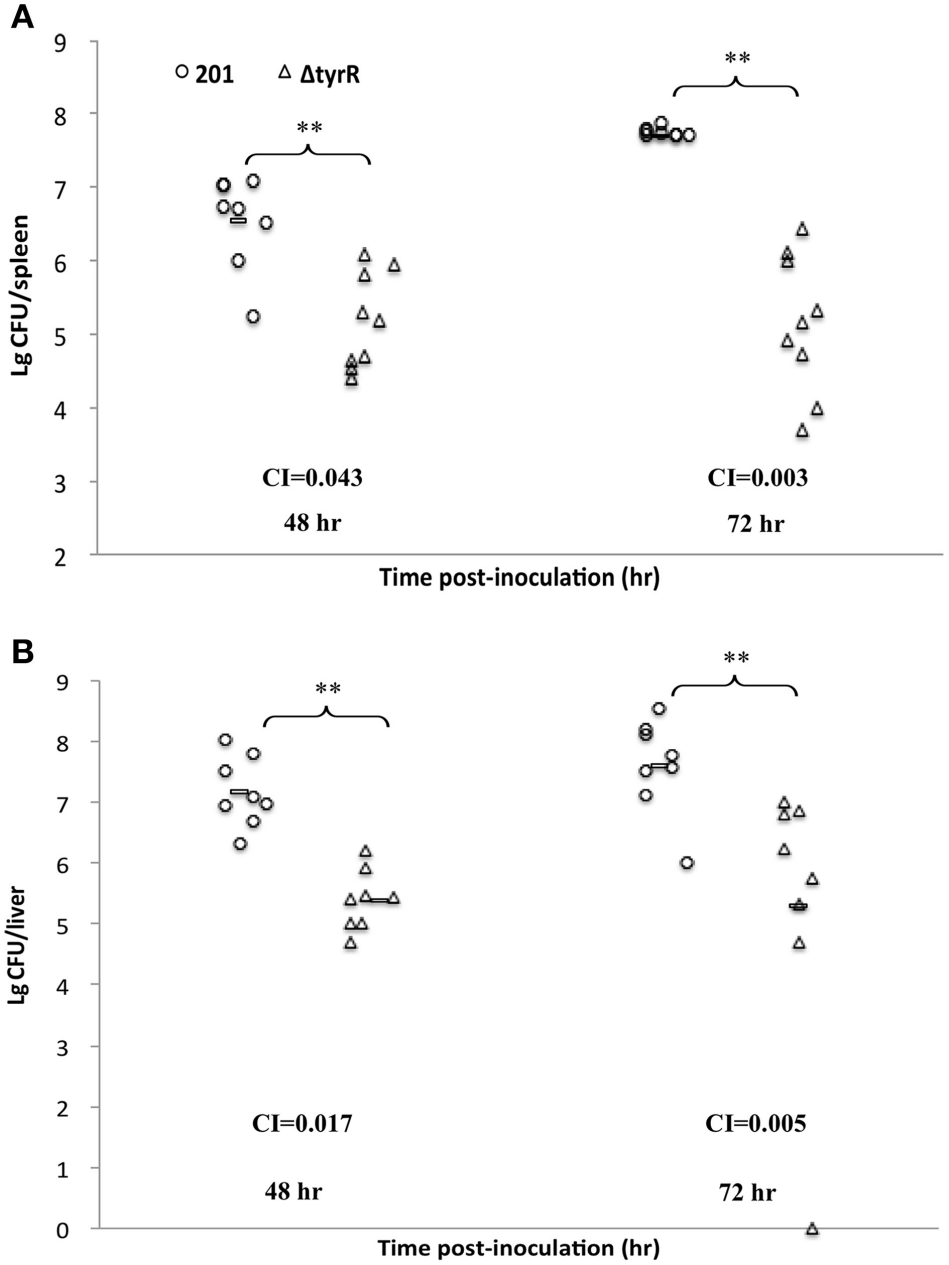
**Growth dynamics of *Y. pestis* upon inoculation by intraveneous route**. Groups of eight mice were inoculated intravenously with bacterial mixture of *Y. pestis* strain 201 and the *tyrR* mutant. Bacterial loads of WT strain (open circles) and *tyrR* mutant (open triangles) in spleen **(A)** and liver **(B)** were determined at 48 h and 72 h post-inoculation. Horizontal bars indicate geometric means. The indicated *P* values were determined using the Student's *t*-test.

Growth of the *tyrR* mutant was not retarded relative to that of WT strain under the condition used *in vitro* (Figure 2), indicating that the mutations do not cause any defect in the growth ability. Therefore, the differences in virulence could be due to the specific involvement of this protein under *in vivo* conditions. We next determine the kinetics of *in vivo* growth to examine the fitness of *Y. pestis* WT and *tyrR* mutant. CI assays were performed by intravenously inoculating the bacterial mixture into mice. The results showed that load burden of WT strain can achieved 10^7^ CFU in the spleen or liver after infection for 48 and 72 h. However, compared with the WT strain, a relatively lower amount bacteria of the *tyrR* mutant strain was recovered from the spleen or liver. The mean CI values in the spleens were 0.043 at 48 h and 0.003 at 72 h (Figure 3A), and similar CI values were obtained in the livers (0.017 at 48 h and 0.005 at 72 h) (Figure 3B). Clearly, the *tyrR* mutant was nearly overwhelmed by the population of WT *Y. pestis in vivo*, indicating that this mutant was much less competitive *in vivo* than its parental strain.

### Identification of differential genes regulated by TyrR

To obtain a representative image of TyrR protein in affecting gene expression throughout growth, we recovered RNA from cultures *in vitro* at different time points to determine the optimal time points. The abundance of the *tyrR* transcript was measured by qPCR (data not shown). The bacterial culture grown in BHI at 26°C for 9 h and then transferred to 37°C for 3 h was chosen for RNA recovery for RNA sequencing.

In total, 29 genes were shown differentially expressed between WT and *tyrR* mutant strain (Table 1). Of these genes, 11 were upregulated and 18 were downregulated in *tyrR* mutant compared with the WT strain. Intriguingly, approximately 45% (13/29) of the differentially regulated genes were derived from three plasmids (pCD1, pMT1, and pCRY), thereby suggesting that the laterally acquired genetic elements might be recruited to TyrR regulon during the process of evolution. Of which, five genes encoding type III secretion proteins in plasmid pCD1 (*yscB*, *yscN*, *yscP*, *sycE*, and *lcrV*) were downregulated 2.0–4.2- fold in *tyrR* mutant relative to WT strain.

**Table 1:** **List of differentially regulated genes in *tyrR* mutant compared to WT strain**.

**Gene ID**	**Gene name**	**Location**	**Start**	**End**	**Strand**	**Fold change(Δ*tyrR*/WT)**		**Annotation**
						**RNA-seq**	**qPCR**	
YP_0398	*aroF*	Chromosome	421761	422837	+	39.5	52.0	Phospho-2-dehydro-3-deoxyheptonate aldolase
YP_0399	*tyrA*	Chromosome	422850	423971	+	19.4		Bifunctional chorismate mutase/prephenate dehydrogenase
YP_0720	*aroL*	Chromosome	777603	778127	+	4.7		Shikimate kinase II
YP_pMT067		pMT1 plasmid	56720	56986	−	3.3		Hypothetical protein
YP_0263	*aroP1*	Chromosome	265038	266438	−	2.9	3.0	Aromatic amino acid transport protein
YP_pCRY14	*virB9*	pCRY plasmid	10302	11210	+	2.9		Type IV secretory pathway VirB9 component
YP_1999	*ompW*	Chromosome	2221841	2222488	+	2.7	1.9	Outer membrane protein W
YP_0929	*tyrP*	Chromosome	999685	1000893	+	2.7	2.3	Tyrosine-specific transport protein
YP_3227	*sirA2*	Chromosome	3601701	3601955	+	2.2		Cell developmental protein SirA
YP_3287		Chromosome	3665321	3665497	−	2.2		Hypothetical protein
YP_3140	*rbsB8*	Chromosome	3507546	3508502	+	2.1		Putative periplasmic protein precursor
YP_0510	*efp1*	Chromosome	546626	547192	+	−2.0		Elongation factor P
YP_pCD52	*lcrV*	pCD1 plasmid	37960	38934	+	−2.0		V antigen LcrV
YP_0029	*rbn*	Chromosome	37669	38613	+	−2.2		rbn; ribonuclease BN; K07058
YP_pMT068		pMT1 plasmid	56986	57930	−	−2.2		Hypothetical protein (exonuclease)
YP_2475	*hdeB*	Chromosome	2747607	2747936	−	−2.3	−3.7	Acid-resistance protein
YP_pMT011		pMT1 plasmid	13338	13673	−	−2.5		Putative phage tail protein
YP_0023	*glnG*	Chromosome	30204	31616	−	−2.6		Nitrogen regulation protein NR(I)
YP_pCD32	*yscB*	pCD1 plasmid	23132	23545	−	−2.8	−4.3	Type III secretion protein SctB
YP_pCD43	*yscN*	pCD1 plasmid	31370	32689	−	−3.2		Type III secretion system ATPase
YP_0045	*rph*	Chromosome	57315	58031	+	−3.5		Ribonuclease PH
YP_0024	*glnH*	Chromosome	31624	32673	−	−3.6		Nitrogen regulation protein NR(II)
YP_pMT032		pMT1 plasmid	31098	31796	−	−3.7		Hypothetical protein
YP_pCD82	*sycE*	pCD1 plasmid	57327	57719	+	−3.8		Putative YopE chaperone SycE
YP_2910	*hdeD*	Chromosome	3232385	3232954	−	−3.9	−6.1	Acid-resistance membrane protein
YP_pCRY17		pCRY plasmid	13516	13914	+	−3.9		Hypothetical protein
YP_pCD41	*yscP*	pCD1 plasmid	29542	30909	−	−4.2		Putative type III secretion protein YscP
YP_pMT031		pMT1 plasmid	30430	31131	−	−5.1		Hypothetical protein
YP_pMT030		pMT1 plasmid	29795	30493	−	−10.6		Putative ABC transporter ATP-binding protein

**Figure 4:**
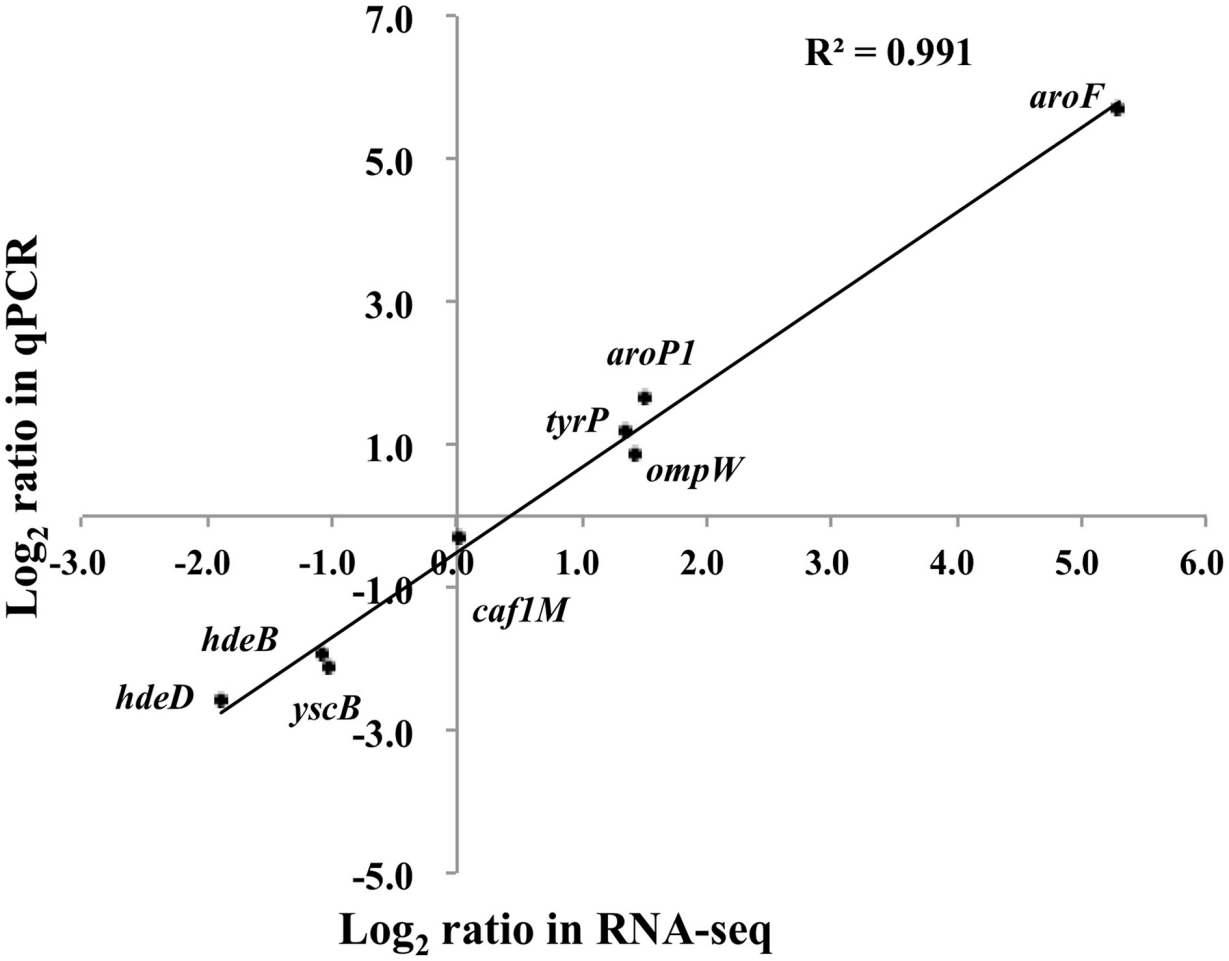
**Transcription measurements of eight genes were chosen for quantitative RT-PCR validation**. The real-time PCR log_2_ values were plotted against the RNA-seq data log2 values. The coefficient of determination (*R*^2^) for comparison of the two datasets is 0.991.

Eight genes were selected for qPCR analysis to validate the RNA-seq data. A high correlation (*R*^2^ = 0.988) was observed between expression values obtained by RNA-seq and qPCR (Figure 4). Aromatic-pathway genes were confirmed to be regulated by TyrR in *E. coli* (Pittard et al., 2005). As expected, the *aroF-tyrA* operon responsible for aromatic biosynthesis and *aroL*, *aroP* and *tyrP* for aromatic transport were most strongly upregulated. Two genes responsible for acid stress, *hdeB* and *hdeD*, were confirmed to be downregulated upon deletion of *tyrR* gene, and this finding was consistent with the observation on the compromised tolerance to acid stress in *in vitro* assays (Figure 1).

### Use of conserved TyrR motif to identify direct TyrR -regulated genes

DNA-binding regulator usually regulates its target genes by directly binding to a dyad DNA consensus sequence. To predict which genes identified by RNA-seq might be under the direct control of TyrR, we extracted the putative TyrR-binding motif sequence (TGTAAA-N_6_-TTTACA) from RegulonDB and conducted a research in the upstream 300 nt of the differentially regulated genes identified by RNA-seq experiment. Four genes were identified as candidates for direct TyrR regulation. As expected, conserved DNA motifs were found in the promoter region of three known aromatic-pathway transcripts (*aroF*, *tyrP*, and *aroP1*). Another candidate targeted by TyrR is the *hdeD* gene, which encodes an acid stress membrane protein, because TyrR box was found ~140 nt upstream of the translational start site of *hdeD*.

### Synthesis of secreted V antigen not influenced by TyrR in *Y. pestis*

*Y. pestis* LcrV is secreted via T3SS machinery during infection and can be exploited as a protective antigen known as V antigen (Skrzypek and Straley, 1995). Abrogation of LcrV expression render *Y. pestis* avirulent (Burrows, 1956). Since LcrV protein is responsible for extracellular survival and dissemination in *Y. pestis*, the down-regulation of *lcrV* might lead to the reduced capability of organ colonization. *Y. pestis* V antigen is maximally expressed and secreted under calcium-restricted conditions at 37°C *in vitro*. Therefore, we compared the levels of secreted V antigen synthesis by using Western blot method in WT and *tyrR* mutant grown in calcium-deficiency TMH medium at 37°C. Unexpectedly, the results showed that the expression of V antigen was not obviously changed upon the deletion of *tyrR* (Supplementary Figure 1). TyrR might have failed to affect LcrV expression at least in *Y. pestis* grown *in vitro*.

## Discussion

Aromatic amino acid biosynthesis is required for the intracellular replication of *Listeria monocytogenes* and *Shigella flexneri* (Cersini et al., 1998; Stritzker et al., 2004). Coincidently, two aromatic-pathway genes (*aroE* and *aroA*) play a crucial role in *Y. pestis* fitness in deep tissues during infection (Palace et al., 2014). *Y. pestis* lacking *tyrR* was significantly less virulent than the WT strain, and complementation of *tyrR* restored WT virulence in the deletion strain, thereby suggesting that TyrR plays an important role in *Y. pestis* virulence. The regulation of aromatic amino acid metabolism has been assigned to the transcription factor, TyrR. This is the first report to demonstrate the effect of this protein on *Y. pestis* pathogenesis. TyrR is not required for growth *in vitro*, but is required for full virulence of *Y. pestis*. Whether the availability of aromatic amino acids *in vivo* might have an effect during the process of infection unknown. Intriguingly, we observed that *tyrR* mutant efficiently protects mice against *Y. pestis* infection as an attenuated strain (data unpublished). The potential of TyrR as a new therapeutic target is worth exploring.

When *Y. pestis* enters invasion sites, most of the bacteria are engulfed and killed by the polymorphonuclear leukocytes (PMNs) that are attracted to these invasion sites. However, a few bacilli survive and proliferate within phagolysosomes of tissue macrophages during the initial infection stage and are then released and to elicit the systematic infection (Perry and Fetherston, 1997). The growth defect of *Y. pestis tyrR* mutant in BHI with pH 5.2 (Figure 1) was a hint that TyrR might be associated with intracellular replication, because acidic pH is a hypothesized prevailing condition in phagolysosomal microenvironments.

Based on the results of RNA-seq-based transcriptional profiling, less than 1% of *Y. pestis* genes were affected by TyrR, thereby indicating that this protein is likely to function as a local regulator. However, it was unexpected that a regulatory defect in a minority of non-essential genes has as much effect on pathogenesis *in vivo* as the removal of a single critical virulence factor. We supposed that the regulation of T3SS encoded by pCD1 plasmid might partially account for the virulence variations between WT and *tyrR*-deletion strains. However, the pCD1 plasmid-encoded genes are downregulated ranging from 2.0 to 4.2 fold, which are generally much lower than that for the aromatic amino acid metabolism genes (2.7 ~ 39.5 fold). In addition, no predicted TyrR-binding sites exist in the regulated genes of T3SS, indicating perhaps TyrR weakly affects transcription of these genes due to lacking exact DNA binding motifs on these plasmids. Thus, we speculated that regulation of T3SS by TyrR is likely to be indirect.

### Conflict of interest statement

The authors declare that the research was conducted in the absence of any commercial or financial relationships that could be construed as a potential conflict of interest.
