# Supplementary figures and images for: TyrR, the regulator of aromatic amino acid metabolism, is required for mice infection of Yersinia pestis
Source: Front Microbiol. 2015 Feb 12;6:110. doi: 10.3389/fmicb.2015.00110 (PMC4325908; doi:10.3389/fmicb.2015.00110)

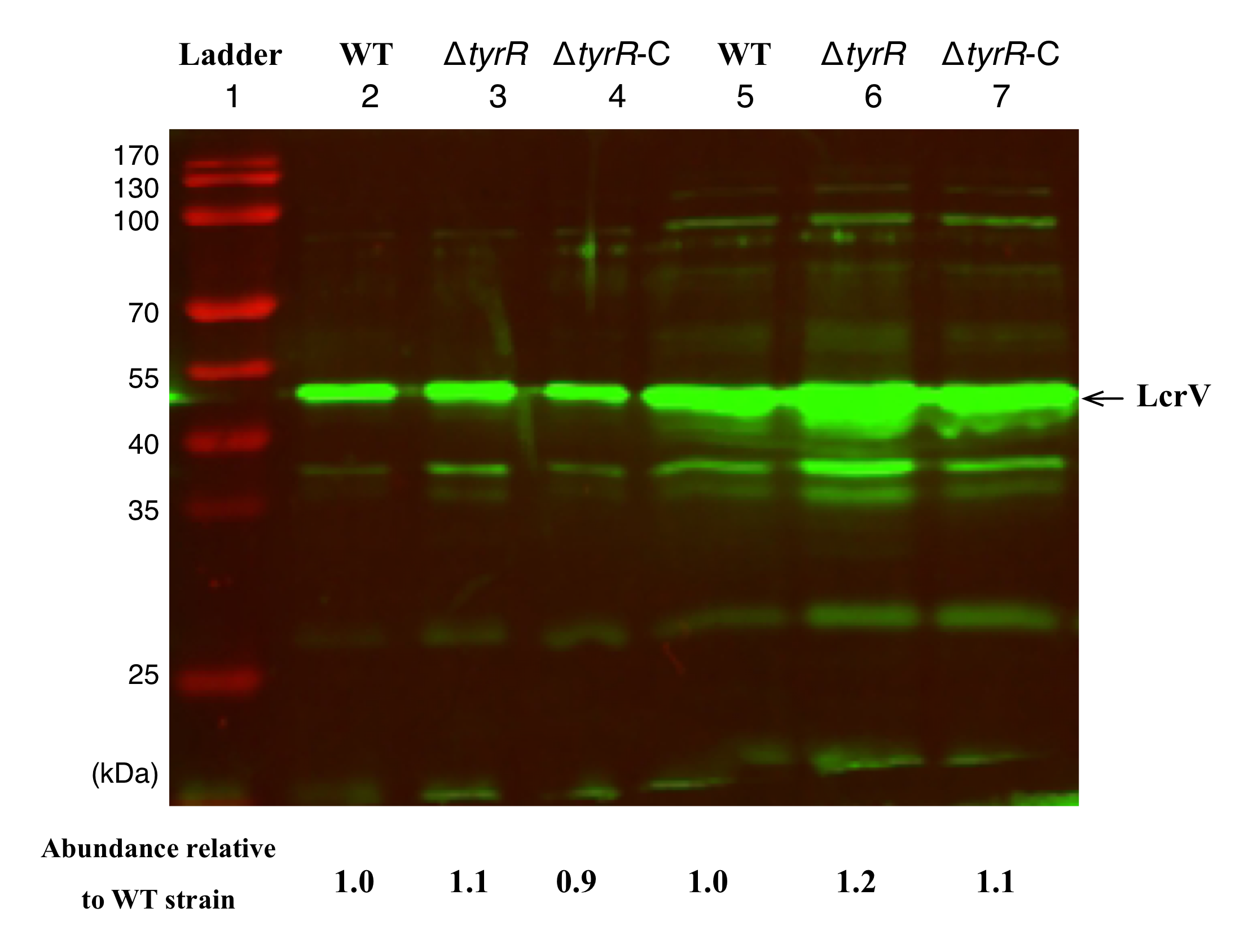

Supplement: Supplementary file 1 [file Image1.TIFF]
